# Research Note: Overview of fowl *adenovirus* serotype 4: structure, pathogenicity, and progress in vaccine development

**DOI:** 10.1016/j.psj.2024.103479

**Published:** 2024-01-30

**Authors:** Iman Pouladi, Hamideh Najafi, Amin Jaydari

**Affiliations:** ⁎ Department of Microbiology and Immunology, Faculty of Veterinary Medicine, University of Tehran, Tehran, Iran; † Department of Microbiology and Food Hygiene, Faculty of Veterinary Medicine, Lorestan University, Khorramabad, Iran

**Keywords:** Fowl *adenovirus* serotype 4, (FAdV), structure, pathogenicity, vaccine

## Abstract

Fowl *adenovirus* serotype 4 (**FAdV**) is highly pathogenic and lethal to chickens, especially broilers, which has emerged as one of the most important economic losses for the poultry industry in the past few years. Although inactivated vaccines have been widely used to control FAdV diseases, with the passage of time and the advancement of technology, live attenuated vaccines and subunit vaccines have also been developed, which are more attractive and effective vaccine candidates. This is an overview of avian *adenoviruses,* especially FAdV, which is related to the structure, pathogenicity of *adenoviruses* in birds, development and strategies used to make and use vaccines using different methods. As well as during this study it was determined that various vaccines against the new FAdV-4 genotype have been developed and many advances have been made in control disease However, many studies conducted in this field need extensive investigation.

## INTRODUCTION

*Adenoviruses* infect a wide range of host species from fish to mammals (Pan et al., 2021). Avi *adenoviruses* are widely distributed in commercial herds worldwide and show a wide range of virulence and clinical symptoms. Some strains of *adenovirus* in birds lead to a severe reduction of lymphocytes in the bursa of Fabricius, thymus, and spleen, which will reduce immunity (Pan et al., 2017; Wang et al., 2022). Fowl *adenovirus* serotype 4 (**FAdV**) is highly pathogenic for chickens, especially 3- to 5-wk-old broilers, and has emerged as one of the most important economic losses for the poultry industry in the last 30 yr. As a result, detection of avian *adenoviruses*, such as Fowl *adenoviruses* (**FAV**), turkey hemorrhagic enteritis virus (**HEV**) and egg drop syndrome (**EDS**) virus, has significant effects on the economic performance of commercial flocks (Hess et al., 2000). Infections caused by avian *adenovirus* (**AAV**) are associated with a of clinical symptoms, including inclusion body hepatitis (**IBH**) and hepatitis-hydro pericardium syndrome (**HHS**), respiratory disease, necrotizing pancreatitis, proventricular Dilatation disease, and gizzard erosion (**GE**). Chickens infected by IBH/HHS show high mortality rate, liver damage and hydro pericardium (Pan et al., 2017; Ruan et al., 2018; Pan et al., 2021). Since Avi *adenoviruses* (**AAV**) infections commonly occur under commercial conditions, several effective strategies have been implemented to control immunosuppressive diseases such as infectious bursal disease virus (**IBDV**) and Chicken anemia virus (**CAV**) that can predispose or enhance AAV pathogenicity (Pan et al., 2021). Currently, several types of vaccines have been developed and evaluated for the control of HHS. Due to the *adenovirus* ability of exogenous genes transferring, natural non-pathogenic FAdV-4 and synthetically attenuated FAdV-4 have created efficient vaccine vectors that protect chickens against HHS and significantly reduce the cost of the combined vaccine (Liu et al., 2022). Also, inactivated vaccines have been used in broiler breeders flocks to prevent vertical transmission, induce the production of maternal antibodies to protect chickens against AAV infection and clinical IBH/HHS (Li et al., 2017; Pan et al., 2017; Tian et al., 2020). Based on this, the present study deals with the pathogenicity of FAdV and the development of functional vaccines using inactivated viruses, live attenuated viruses and subunit vaccines, against the main pathogen of the poultry industry, especially the new FAdV-4.

## MATERIALS AND METHODS

### Search Strategy and Data Extraction

Our search of MEDLINE included PubMed, Scopus, Science Direct, Web of Science (ISI), Google Scholar (as English databases); using the following terms: AAV, FAdV, structure, pathogenicity, *adenovirus*, vaccines, inactivated viruses, live attenuated viruses, subunit antigens, and combination vaccines. An extensive search of published and unpublished articles, as well as abstracts and summaries of parasitology congresses, was conducted to gather precise information. English articles were consulted for data collection. A protocol for data extraction was defined and assessed independently.

### Study Selection and Identification

During this study, we searched and identified 315 articles related to the design and production of practical vaccines against FAdV. After removing 60 duplicates, a total of 155 studies were retrieved, 55 of which were rejected solely based on titles and 78 after reviewing abstracts. Consequently, 65 studies were reviewed based on the inclusion and exclusion criteria and evaluated for eligibility. Ultimately, 25 studies met the eligibility criteria and were included in the final systematic review.

## RESULTS AND DISCUSSION

### The Classification and Structure of Fowl Adenovirus

Currently, the *Adenoviridae* family is divided into 5 genera. Members of the genera Mast *adenovirus* and Avi *adenovirus* infect mammals and birds, respectively. The genera At *adenovirus* and Si *adenovirus* have wider host ranges and each includes birds. The fifth genus of the *Adenoviridae* family includes Icht *adenovirus*. The genus Avi *adenovirus* includes only *adenoviruses* (**AdVs**) of avian origin (Harrach et al., 2011; Pan et al., 2021). FAdVs are divided into 5 species (FAdV-A, FAdV-B to FAdV-E) and 12 serotypes, which are grouped as follows: Fowl *Adenovirus* A (**FAdV-1**), Fowl *Adenovirus* B (**FAdV-5**), Fowl *Adenovirus* C (**FAdV-4**) and (**FAdV-10**) Fowl *Adenovirus* D (**FAdV-2, FAdV-3, FAdV-9** and **FAdV-11**), Fowl *Adenovirus* E (**FAdV-6, FAdV-7, FAdV-8a** and **FAdV-8b**). FAdV infections are associated with hepatitis, HHS and gizzard erosion in chickens and other birds, most of which HHS is caused by FAdV serotype 4 (**FAdV-4**) (Harrach et al., 2011; Ruan et al., 2018). In addition to FAdVs, other species of the genus avi *Adenoviruses* have been isolated or detected in turkeys, geese, ducks, pigeons, and various species of falcons. Goose *adenovirus* A (probably 5 types, goose *adenovirus* 1–5), turkey *adenovirus* B (*adenovirus* type turkey 2 and 1) belong to the genus Avi *adenovirus*. Pigeon *adenovirus* A (pigeon *adenovirus* type 1) and duck *adenovirus* B (duck *adenovirus* type 2) also belong to genus (Harrach et al., 2011). *Adenoviruses* have a nonenveloped capsid and icosahedron symmetry. The diameter of the capsid is about 90 nm. The capsid shell consists of 240 capsomeres, which include pentons and hexons. The 12 pantones located at each of the vertices have a pantone base and a fiber. hexons form the faces of the icosahedron capsid and pentons cover the vertices of the capsid. Two morphologically unique antenna-like fiber proteins anchor each penton base. Binding proteins are minor capsid proteins (pIIIa, pVI, and pVIII) that are located on the inner surface. Terminal protein, μ protein, V protein and VII protein bind to the DNA genome in virus particles, which are part of the core proteins. FAdV-4 is grouped in the FAdV-C species and has a linear double-stranded DNA of 35 to 36 kb that encodes about 40 structural and nonstructural proteins. The virus genome has additional terminal sequences that contain inverted terminal repeats (**ITRs**). The terminal protein (**TP**) is covalently attached to each end of the genome (Xie et al., 2013).

### Pathogenesis

**Figure 1:**
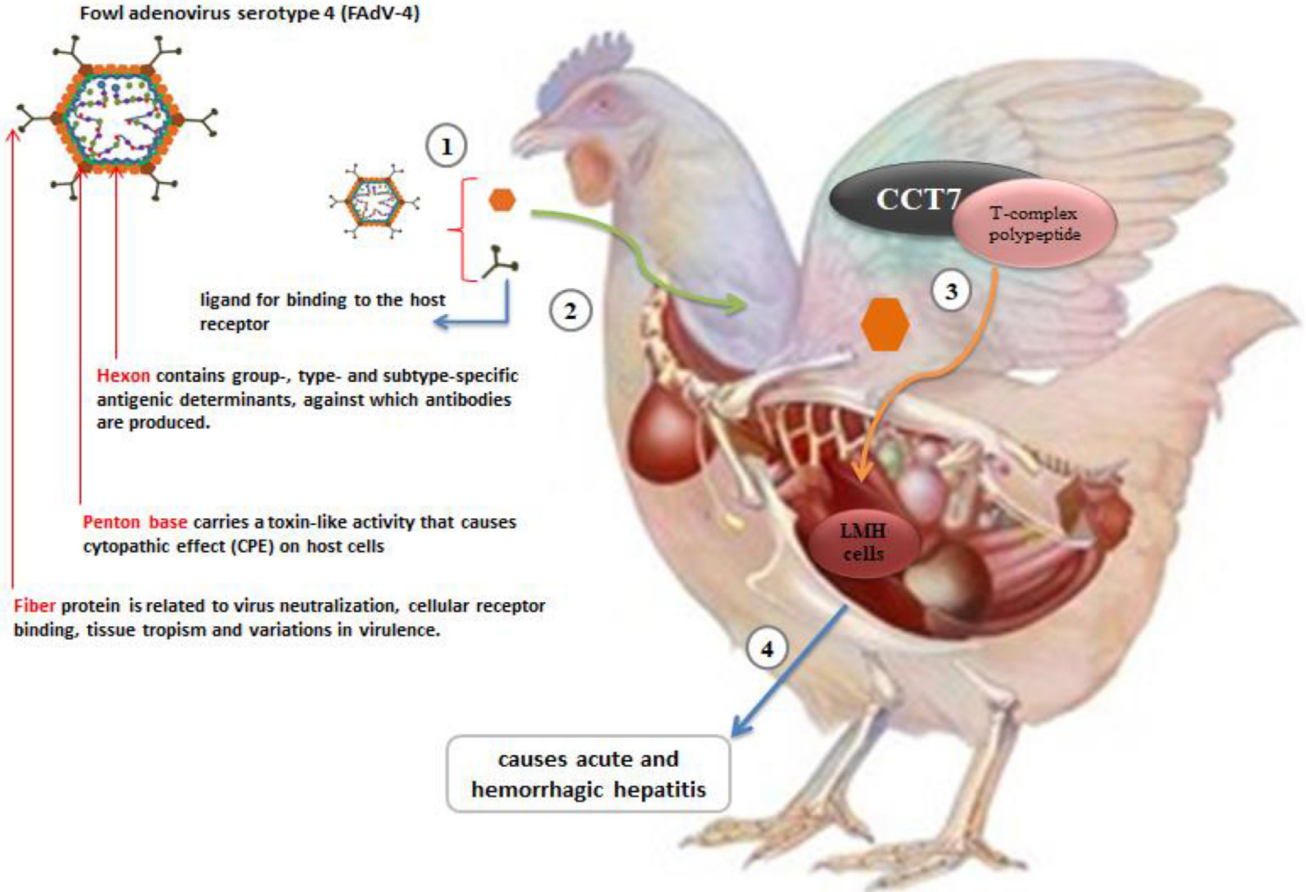
Roles of viral proteins in the pathogenesis of FAdV-4: 1) the increased virulence of hypervirulent FAdV-4 associated with the fiber-2 and hexon genes. 2) Fiber protein consists of an N-terminal tail region, involved in attachment to the penton base; a C-terminal knob region, which has been assumed to be the ligand for binding to the host receptor. 3) Viral proteins can make full use of host factors to facilitate replication and pathogenicity during infection. hexon hijacked T-complex polypeptide 1 subunit eta (CCT7) to contribute hypervirulent FAdV-4 replicating in LMH cells, an immortalized chicken liver cell line. 4) FAdV-4 is a hepatotrophic virus that causes acute and hemorrhagic hepatitis.

The FAdV-4 is the cause of diseases such as nephritis, hepatitis and hydro pericardial syndrome (**HHS**), which is a severe disease in broilers with the accumulation of clear fluid in the pericardial sac. Its mortality rate is between 30 and 100%. The liver is the main target organ for FAdV-4 infections, and chickens infected with the virus usually show signs of hydro pericardial syndrome. This virus is highly contagious and spreads both vertically and horizontally (Li et al., 2017; Liu et al., 2022) (Figure 1). These viruses have a wide distribution. Therefore, *adenoviruses* have been isolated from peripheral blood, bursa, central nervous system, feces, trachea, conjunctiva, pharynx, lung, kidney, liver and spleen. The target tissues were homogenized in 0.01 M sterile PBS (pH 7.4) and frozen at -20°C. Then, to isolate the virus, the homogenates are centrifuged at 8000 rpm for 10 min at 4°C. Supernatants were collected and inoculated into 10-day-old fertilized SPF eggs via the chorioallantoic membrane (**CAM**) inoculation route. After 7 d of incubation at 37°C, the target tissues and CAM of inoculated embryos are used for viral DNA extraction. The virus is shed in the feces for up to 14 d after infection, with peak titers between d 4 and 7. In the terminal ileum, there are viral antigens in some of the columnar and goblet cells of the epithelial layers. The cells are infected at the base of the villus and have migrated to the surface of the villus within 5 to 10 h required for virus replication (Li et al., 2017; Xie et al., 2021a).

### New Vaccines Against Fowl Adenoviruses

In recent years, HHS has been reported from different parts of the world, including the Middle East, Slovakia, India, Russia, Japan, South Korea and China. Its mortality rate is between 30% and 80%. Proper disinfection of facilities and equipment, adequate ventilation, adequate lighting, and limited entry of poultry workers or visitors may be helpful in preventing HHS. However, vaccination against HHS provides broad protection to vaccinated chickens and minimizes direct losses. Therefore, it is the most basic method to control HHS (Li et al., 2017). The new FAdV-4 genotype has spread rapidly and caused huge economic losses and threatened the health of poultry industry (Liu et al., 2022). The histopathological study of the disease shows that the damage of the genotype is mostly seen in the heart, liver, spleen, lung, kidney, and especially the liver, and the expression level of interleukin 1β (**IL-1β**), interleukin 6 (**IL-6**), interleukin 8 (**IL-8**), interferons (**IFNs**) and tumor necrosis factor -α (**TNF-α**) during viral infection increased significantly (Meng et al., 2019; Zhang et al., 2021).

### Inactivated Monovalent Vaccines

Traditional inactivated vaccine immunization is the main preventive method for some poultry diseases, especially emerging viruses. Due to the emergence of the new genotype of hypervirulent FAdV-4, immunization by inactivated vaccines is one of the main methods of prevention, which have advantages such as safety, cost savings, good humoral immune effect and low effect of maternal antibodies (Liu et al., 2022). In the early stages of HHS outbreaks, inactivated vaccines prepared from infected liver homogenate provide the main means of disease control. Although relatively satisfactory results were obtained using these formalin-inactivated vaccines, there were still many concerns about the spread of HHS, due to inadequate inactivation of pathogenic strains, poor sanitary conditions during vaccine production, lack of adequate adjuvant, and inadequate amount of virus (Li et al., 2017; Liu et al., 2022). In order to minimize the risk of HHS spread, the cell culture systems and SPF chickens with strict biosecurity and high hygiene standards are better choices for the development of inactivated vaccines. Recently, an inactivated vaccine similar to emulsion oil not only protects against FAdV-4 in vaccinated chickens, but also provides extensive cross-protection against different FAdV serotypes in vaccinated chickens (Li et al., 2017).

There is concern whether these FAdV-4-containing vaccines alone can protect against malignant IBH caused by other FAdV serotypes. Therefore, during the study of Kim et al. (2014), the cross-protection of an inactivated FAdV-4 oil emulsion vaccine against different serotypes of FAdV environmental isolates was investigated. During their study, it was shown that inactivated FAdV-4 oil emulsion vaccine can provide broad cross-protection against different FAdV serotypes in vaccinated birds. Therefore, it is concluded that the inactivated FAdV-4 oil emulsion vaccine can be effective in preventing the spread of other FAdV serotypes as well as FAdV-4 infection in the poultry industry (Kim et al., 2014).

Another method of making inactivated vaccines is designing and using CRISPR-Cas9-based technique in order to produce a recombinant genetic engineering vaccine against both FAdV-4 and FAdV-8 strains. In the present study, the recombinant FAdV-4 expressing FAdV-8b fiber, designated as FA4-F8b, and the FAdV-8b was produced and expressed by CRISPR-Cas9 and homologous recombination techniques. All these suggest that the inactivated recombinant FA4-F8b could serve as a vaccine candidate for the control of HHS and IBH (Pan et al., 2017) (See Table 1).

**Table 1:** Some inactivated, live attenuated, and subunit vaccines developed against FAdV.

Type of vaccine	Preparation	Immune efficacy	References
Inactivated vaccine	Virus strain HLJFAd15 (The highly pathogenic FAdV-4 strain,GenBank accession number KU991797) was propagated on primary chicken embryo liver (CEL) cells and used for vaccine production and challenge strain. For inactivation of the virus, formaldehyde was added to the virus culture medium, harvested from FAdV-4 infected CEL cells with a concentration of 0.2% in the final product.	The vaccine provided a high level of antibody, preferential T helper 2 (Th2) (interleukin-4 secretion) not Th1 (interferon-γ secretion) response, and full protection against a lethal dose of the novel hypervirulent FAdV-4.	Pan et al., 2017
	Recombinant FAdV-4 expressing the fiber of FAdV-8b, designated as FA4-F8b, expressing fiber of FAdV-8b was generated by the CRISPR-Cas9 and homologous recombinant techniques.	The inoculation of inactivated the FA4-F8b in chickens could not only induce highly neutralizing antibodies, but also provide efficient protection against both FAdV-4 and FAdV-8b.	Lu et al., 2022
	A chimeric FAdV-4 was firstly generated by substituting fiber-1 of FAdV-4 with fiber of FAdV-8b. The chimeric virus, rFAdV-4-fiber/8b, exhibited similar replication ability in vitro and pathogenicity in vivo to the parental wild type FAdV-4.	A single dosage of vaccination with the inactivated rFAdV-4-fiber/8b induced high antibody titers against fiber-2 of FAdV-4 and fiber of FAdV-8b and provided full protection against FAdV-4 and -8b challenge.	Wang et al., 2022
	Developed a novel inactivated oil-adjuvanted vaccine derived from rHN20 strain and evaluated its immunogenicity in specific-pathogen-free chickens.	Chickens subcutaneously or intramuscularly immunized with the inactivated vaccine produced high titers of neutralizing antibodies and were protected from a lethal dose of virulent wild-type FAdV-4 challenge.	Zhang et al., 2022
	The virus strain SDJN0105 was propagated on chicken embryo liver (CEL) cells and vaccine production. For inactivation of the virus, formaldehyde was added to the virus culture medium collected from CEL cells infected with FAdV-4 with a 0.2% concentration in the final product.	The vaccine could provide full protection for SPF chickens against a lethal dose of the FAdV-4 strain SDJN0105 and a high level of antibodies.	Meng et al., 2019
Live attenuated vaccine	Used a CRISPR/Cas9-based homology-dependent recombinant technique to replace the fiber-2 gene with egfp and generate a novel recombinant virus, designated FAdV4-EGFP-rF2. Although FAdV4-EGFPrF2 showed low replication ability compared to the wild-type FAdV-4 in LMH cells, FAdV4-EGFP-rF2 could effectively replicate in LMH-F2 cells with the expression of Fiber-2.	FAdV4-EGFP-rF2 was not only highly attenuated in chickens, but also could provide efficient protection against a lethal challenge of FAdV-4. Moreover, FAdV4-EGFP-rF2 without fiber-2 could induce neutralizing antibodies at the same level as FA4-EGFP with fiber-2.	Xie et al., 2022
	The FAdV-4 rHN20 fosmid was generated from the HLJFAd15 strain. Recombinant fosmids were engineered from rHN20 in Escherichia coli DH10B cells using the Counter-Selection BAC Modification kit.	A recombinant rHN20-vvIBDV-VP2 strain, expressing the VP2 protein of very virulent infectious bursa disease virus (vvIBDV), was rescued and showed complete protection against FAdV-4 and vvIBDV and the novel FAdV4 vector could provide sufficient protection for HHS and efficient exogenous gene delivery.	Pan et al., 2021
	The hexon gene is the key gene responsible for the high pathogenicity of FAdV-4 and constructed a non-pathogenic chimeric virus rHN20 strain based on the emerging FAdV-4. The immunogenicity of artificially rescued rHN20 was evaluated in chickens using different routes and doses as a live vaccine.	The live rHN20 vaccine induced high titers of neutralizing antibodies against FAdV-4 and fully protected the immunized chickens against a lethal dose of FAdV-4. Furthermore, immunized chickens showed no clinical symptoms or histopathological changes in the FAdV-4-targeted liver, and the viral load in the tissues of immunized chickens was significantly lower than that of chickens in the challenge control group.	Zhang et al., 2021
	A recombinant virus FA4-EGFP expressing EGFP-Fiber-2 fusion protein was generated by the CRISPR/Cas9 technique. Although FA4-EGFP shows slightly lower replication ability than the wild type (WT) FAdV4, FA4-EGFP was signifcantly attenuated in vivo compared with the WT FAdV-4.	Chickens infected with FA4-EGFP did not show any clinical signs, and all survived to 14 day post-infection (dpi), whereas those infected with FAdV-4 showed severe clinical signs with HHS and all died at 4 dpi. Besides, the inoculation of FA4-EGFP in chickens provided efcient protection against lethal challenge with FAdV-4.	Xie et al., 2021a
	Generated an infectious clone, pFAdV-4 ON1, containing the entire viral genome of a nonpathogenic FAdV-4 (ON1 isolate). PFAdV-4 ON1 was used for targeted deletion of open reading frames (ORFs) 16 and 17 and replacement with the enhanced green fluorescence protein (EGFP) expression cassette to generate recombinant viruses.	Their replication was significantly reduced with respect to that of the wild-type virus. These observations suggest the potential utility of FAdV-4 as a vaccine vector and the importance of ORFs 16 and 17 for virus replication at wild-type levels.	Pei et al., 2018
Recombinant viruses	A chimeric fiber protein retaining epitopes from FAdV-4 and FAdV-11 was designed in this study.	The development of neutralizing antibodies was limited against FAdV-11 and absent against FAdV-4, indicating that protection granted by such an antigen may be linked to different immunization pathways.	De Luca et al., 2022
	Construction of expression plasmid and expression and purification of recombinant fiber-2 protein.	The lowest dose of recombinant fiber-2 protein that could provide 100% protection against challenge with virulent FAdV-4 strain HB1501 as well as elicit protective immunity was 2.5 μg/bird.	Ruan et al., 2018
	The respective open reading frames of fiber-1, fiber-2, penton base, and loop-1 region of hexon were amplified by PCR from viral DNA. PCR products were cut with their respective restriction endonucleases and ligated into the vector, pET32a.	According to challenge mortalities and tissue gross/micro lesion results, fiber-2 induced the best protection, followed by fiber-1 and hexon. Fiber-1 and fiber-2 provided complete protection against 105.5 TCID50 viral load challenge with 100 or 50 μg doses per chicken, respectively. Penton could induce effective protection only at the high dosage of 200 μg per chicken.	Wang et al., 2018
	Three different FAdV-4 derived capsid proteins, fiber-1, fiber-2, and hexon loop-1, were expressed in a baculovirus system and tested for their capacity to induce protection in chickens.	The fiber-2 vaccinated group displayed high resistance againstthe adverse effects of the challenge with only one dead bird out of 28, as compared to the challenge control group where the infection caused 78% mortality. A moderate protective effect resulting in 38% mortality was observed for fiber-1, whereas the hexon loop-1 vaccinated group was not effectively protected as manifested by 73% mortality.	Schachner et al., 2014
	A chimeric fiber protein retaining epitopes from FAdV-4 and FAdV-11, named crecFib-4/11, was designed and expressed.	Immunized birds experienced a significant B cell increase in the liver upon challenge, remaining high throughout the experiment. Furthermore, vaccination stimulated the proliferation of cytotoxic T lymphocytes, with earlier circulation in the blood compared to the challenge control and subsequent increase in liver and spleen.	De Luca et al., 2022
	A subunit vaccine candidate derived from the bacterially expressed recombinant Fiber2 protein (termed as rFiber2 subunit vaccine) of FAdV-4 GZ-QL strain and a recombinant plasmid pVAX1-Fiber2 as DNA vaccine candidate (termed as Fiber2 DNA vaccine).	Three injections of the rFiber2 subunit vaccine or the Fiber2 DNA vaccine induced robust humoral and cellular immune responses in chickens. the efficacy of the rFiber2 subunit vaccine was significantly higher (80 %–100 %) compared with the Fiber2 DNA vaccine (50%–60%) and a commercial inactivated vaccine (80 %).	Yin et al., 2021
	A recombinant NDV LaSota vaccine strain expressing full length fiber-2 gene of FAdV-4 (rLaSota-fiber2) was generated using reverse genetics. The FAdV-4 fiber-2 protein was expressed as a soluble form rather than NDV membrane-anchored form.	Single-dose intramuscular vaccination of 2-week-old SPF White Leghorn chicks with the live or inactivated rLaSota-fiber2 provided complete protection against virulent NDV challenge. Single-dose intramuscular vaccination with the live rLaSota-fiber2 vaccine provided better protection against virulent FAdV-4 challenge and significantly reduced faecal viral shedding comparing to the inactivated vaccine.	Tian et al., 2020
	User-Friendly Reverse Genetics System for Modification of the Right End of Fowl Adenovirus 4 Genome. Three recombinant viruses were constructed to test the assumption that species-specific viral genes of ORF4 and ORF19A might be responsible for the enhanced virulence: viral genes of ORF1, ORF1b and ORF2 were replaced with GFP to generate FAdV4-GFP, ORF4 was replaced with mCherry in FAdV4-GFP to generate FAdV4-GX4C, and ORF19A was deleted in FAdV4-GFP to generate FAdV4-CX19A.	Survival analysis showed that FAdV4-CX19A-infected chicken embryos survived one more day than FAdV4-GFP- or FAdV4-GX4C-infected ones. The results illustrated that ORF4 and ORF19A were non-essential genes for FAdV-4 replication although deletion of either gene influenced virus growth.	Yan et al., 2020

### Live Attenuated Vaccines

During recent studies, inactivated vaccines, subunit vaccines and genetically engineered vaccines have been reported for FAdV-4 strain. In contrast, live Attenuated FAdV-4 vaccines have not been extensively studied. Live vaccines are made based on low pathogenic or nonpathogenic strains (Table 1). To date, 3 naturally occurring nonpathogenic FAdV-4 strains have been isolated: strain ON1 isolated from Canada, strain KR5 isolated from Japan, and strain B1-7 isolated from India. However, the protective efficacy of these strains against FAdV-4 is unclear (Zhang et al., 2021). Recent studies have shown that recombinant viruses constructed from the strain FAdV-4 new genotype with enhanced green fluorescent protein (**EGFP**) fused to the N-terminus of the fiber-2 gene or in which the N-terminus of fiber-2 is deleted, is not pathogenic for specific pathogen-free (**SPF**) chickens, but the titer of recombinant viruses in vitro was significantly lower than that of wild-type FAdV-4. Live vaccines show characteristics similar to natural infection by the virus and allow the immune system to develop strong immunity to prevent disease (Pan et al., 2021; Xie et al., 2021a).

There are several reports of the use of live attenuated vaccines against FAdV-4, where virus attenuation was achieved by serial passage of the virus in chicken embryos or QT-35 cells. SPF chickens vaccinated with FAdV-4 ON1 strain orally and by intramuscular injection showed a strong anti-FadV-4 specific antibody response, a VN 1:50 antibody responses, increased expression of interferon IFN-γ and interleukin IL-10 in liver, and decreased the expression of IFN-γ and IL-18 in the spleen (Li et al., 2017; Liu et al., 2022).

The present research and development of FAdV-4 vaccine is mainly focused on inactivated vaccines and relatively less on live vaccines, and accordingly, in the study of Yu Zhang et al. (2021), rescued immunogenicity of synthetic nonpathogenic chimeric virus strain rHN20 -as a live vaccine- in chickens with the use of different routes and doses was evaluated. The live rHN20 vaccine induced high titers of neutralizing antibodies against FAdV-4 and fully protected immunized chickens against a lethal dose of FAdV-4 (Zhang et al., 2021).

Today, updated and advanced techniques and mechanisms are used for the development of antiviral vaccines in poultry, such as using the CRISPR/Cas9-based technique to develop and make attenuated live vaccines against avian adenovirus serotype 4. For example, the studies conducted in this field have used a FA4-EGFP recombinant virus expressing EGFP-Fiber-2 fusion protein with CRISPR/Cas9 technique. During the present study, chickens infected with FA4-EGFP showed no clinical signs and all survived up to 14 days post-infection, while chickens infected with FAdV-4 showed severe clinical signs with HHS and all died. Compared to an inactivated vaccine, FA4-EGFP induced higher titer neutralizing antibodies earlier (Xie et al., 2022). Also targeting the -N terminal of Fiber-1 gene and producing a recombinant virus FAdV4-RFP_F1 that expresses RFP and Fiber-1 fusion protein based on CRISPR/Cas9 technique, all this suggests that recombinant FAdV4-RFP_F1 virus can serve as an efficient live attenuated vaccine for FAdV-4 (Lu et al., 2022).

Another study that used the CRISPR-Cas9-based technique for vaccine production is the creation of a FAV-4_Del virus edited with fiber-2 by removing amino acids 7 to 40 in fiber-2 through the CRISPR-Cas9 technique. It was shown that inoculation of FAV-4_Del in chickens can confer complete protection against the lethal wild-type FAdV-4 virus. All these findings not only provide new insights into the molecular basis for FIB-2 pathogenesis, but also provide efficient purposes for the development of antiviral strategies and live attenuated vaccine candidates against hypervirulent FAdV-4 (Xie et al., 2021b) (See Table 1).

### Recombinant Vaccines

Several recombinant vaccines against Fowl *Adenovirus* 4 have been developed. In general, the recombinant vaccines that were investigated in this study are classified into subunit vaccines, viral vector-based vaccines, plasmid DNA vaccines, and reverse genetic vaccines (Table 1).

In a study by Carlotta De Luca et al. (2020), the efficacy of a recombinant FAdV fiber protein, derived from a FAdV-8a strain, was tested to protect chickens against hepatitis (IBH). FAdV-E field isolates belonging to both homotypic (**FAdV-8a**) and heterotypic (FAdV-8b) serotypes were used as challenges. The birds were clinically protected against the homologous challenge virus and showed a significant reduction of the viral load in the target organs examined, while the fiber-based immunity failed to counteract the infection of the heterologous serotype. In protected birds, fiber vaccination prevented the loss of peripheral B cells in the blood after challenge (De Luca et al., 2020).

The studies in which the crecFib-4/11 recombinant chimeric fiber proteins were used to produce subunit vaccines in order to protect chickens against HHS caused by avian *adenovirus* serotype 4, showed that the vaccine containing chimeric fiber protein of recombinant FAdV played a key role in protecting chickens against HHS, and also prevented the emergence of clinical symptoms and viral load in the liver, spleen and bursa of Fabricius, compared to challenge control (Wang et al., 2018; De Luca et al., 2022). According to the results of the study by Schachner et al. (2014) and Ruan et al. (2018), it was determined that recombinant fiber-2 protein acts as a protective immunogen and it can induce 100% protection against the dangerous FAdV-4 HB1501 strain, and the chickens do not have any clinical symptoms and lesions. For this reason, the use of recombinant fiber-2 protein is suggested as a suitable candidate for subunit vaccine to prevent hepatitis-hydro pericardium syndrome caused by fowl adenovirus serotype 4 in chickens (Schachner et al., 2014; Ruan et al., 2018).

The present study has discussed on the pathogenicity of serotype 4 *adenoviruses* in poultry and the development of vaccines using different methods such as the use of inactivated viruses, live attenuated viruses, subunit antigens and recombinant vaccines against the new genotype FAdV-4, which is one of the main pathogens of the poultry industry, and so during this study it was found that different vaccines against the new FAdV-4 genotype have been developed and many advances have been made in the control of emerging HHS, however, many studies conducted in this field need extensive review.

Traditional inactivated vaccine immunization is the main preventive method for some poultry diseases, especially emerging viruses. In the inactive vaccine derived from the conventional FAdV-4 genotype, which conducts cross-protection with other FAdV serotypes, the cross-protection caused by the new FAdV-4 genotype needs a systematic and extensive analysis in different serotypes. Also, considering that relatively satisfactory results were obtained using inactivated vaccines, there are still many concerns about the spread of HHS, due to inadequate inactivation of pathogenic strains, poor sanitary conditions during vaccine production, lack of appropriate adjuvant and inadequate amount of virus. In contrast, the live FAdV-4 vaccines have not been yet extensively studied. Live vaccines are made based on low pathogenic or nonpathogenic strains. To date, 3 natural nonpathogenic FAdV-4 strains have been isolated. However, the protective efficacy of these strains against FAdV-4 is unclear. Live vaccines exhibit characteristics similar to natural infection by the virus, allowing the immune system to induce strong immunity to prevent disease. Also, according to the previous studies, Pantone-based subunit vaccines and fiber-2 recombinant proteins provide high protection. Therefore, they can be attractive candidates for vaccine production to prevent the spread of flow *adenovirus* serotype 4 in chickens. Today, updated and advanced techniques and mechanisms are available for the development of antiviral vaccines in poultry, such as the use of CRISPR/Cas9-based techniques for the development and production of attenuated live and inactivated vaccines against flow *adenovirus* serotype 4, that can be used in the future as a suitable replacement for old vaccines and a suitable method to prevent widespread outbreaks of poultry viral diseases, especially flow *adenovirus* serotype 4. On the other hand, some studies show that research is limited seriously to things like new adjuvants used in new vaccines such as DNA or mRNA-based vaccines against the new FAdV-4 genotype. Thus in order to control infections caused by avian *adenovirus* (**AAV**) which is occurred with a wide range of clinical symptoms including IBH and Hydr opericardial Syndrome caused by the new hyper virulent FAdV-4 genotype, more research is needed in the field of making and developing effective vaccines such as recombinant vaccines using techniques based on reverse genetic vaccines and CRISPR/Cas9.
